# Relationship between placental pathology and neonatal outcomes

**DOI:** 10.3389/fped.2023.1201991

**Published:** 2023-06-15

**Authors:** Xiaojun Guo, Yixiao Wang, Hong Yu

**Affiliations:** Department of Obstetrics and Gynecology, Southeast University Affiliated Zhongda Hospital, Nanjing, China

**Keywords:** preterm birth, preterm premature rupture of membranes, acute intrauterine infection/inflammation, maternal placental vascular perfusion, neonatal outcomes

## Abstract

**Objective:**

To evaluate the relationship between maternal vascular malperfusion and acute intrauterine infection/inflammation with neonatal outcomes.

**Methods:**

This was a retrospective study of women with singleton pregnancies who completed placenta pathological examination. The aim was to study the distribution of acute intrauterine infection/inflammation and maternal placental vascular malperfusion among groups with preterm birth and/or rupture of membranes. The relationship between two subtypes of placental pathology and neonatal gestational age, birth weight Z-score, neonatal respiratory distress syndrome, and intraventricular hemorrhage was further explored.

**Results:**

990 pregnant women were divided into four groups, including 651 term, 339 preterm, 113 women with premature rupture of membranes, and 79 with preterm premature rupture of membranes. The incidence of respiratory distress syndrome and intraventricular hemorrhage in four groups were (0.7%, 0.0%, 31.9%, 31.6%, *P* < 0.001) and (0.9%, 0.9%, 20.0%, 17.7%, *P* < 0.001), respectively. The incidence of maternal vascular malperfusion and acute intrauterine infection/inflammation were (82.0%, 77.0%, 75.8%, 72.1%, *P* = 0.06) and (21.9%, 26.5%, 23.1%, 44.3%, P = 0.010), respectively. Acute intrauterine infection/inflammation was associated with shorter gestational age (adjusted difference −4.7 weeks, *P* < 0.001) and decreased weight (adjusted Z score −2.6, *P* < 0.001) than those with no lesions in preterm birth. When two subtype placenta lesions co-occurrence, shorter gestational age (adjusted difference −3.0 weeks, *P* < 0.001) and decreased weight (adjusted Z score −1.8, *P* < 0.001) were observed in preterm. Consistent findings were observed in preterm births with or without premature rupture of membranes. In addition, acute infection/inflammation and maternal placenta malperfusion alone or in combination were associated with an increased risk of neonatal respiratory distress syndrome (adjusted odds ratio (aOR) 0.8, 1.5, 1.8), but the difference was not statistically significant.

**Conclusion:**

Maternal vascular malperfusion and acute intrauterine infection/inflammation alone or co-occurrence are associated with adverse neonatal outcomes, which may provide new ideas for clinical diagnosis and treatment.

## Introduction

Preterm birth is a common cause of neonatal morbidity and mortality, accounting for about 12 percent of all newborns (1–3). A fully functioning placenta can provide newborns with essential materials, and loss of function can lead to pregnancy complications (4). Placental microscopy and histopathological features are of great significance in explaining the etiology of preterm birth and may also be one of the new methods of disease classification (5–7). The Amsterdam classification system defines four patterns of placental pathology for placental injuries, including maternal vascular malperfusion (MVM), acute intrauterine infection/inflammation (AI), fetal vascular malperfusion (FVM), and chronic inflammation (CI) of unknown etiology (8). F Arias et al. found that MVM and AI are two important placental pathological subtypes of preterm birth and preterm premature rupture of membranes (PPROM) (9).

Elisabeth B et al. found that AI was associated with necrotizing enterocolitis (NEC) in extremely preterm infants (< 28 weeks) (OR12.2 95%CI 1.1, 137.1), and MVM was associated with decreased fetal birth weight (10). Other studies have shown that MVM can lead to more severe bronchopulmonary dysplasia in newborns (11, 12), And might be associated with an increased risk of neurodevelopmental abnormalities in children at 2 years old (13). Co-occurrence of MVM and AI may be a specific form of placental lesions associated with RDS and IVH in preterm birth (14). However, many references exist about the relationship between placental lesions and adverse outcomes. There is still little information on the occurrence of single or combined placental lesions in pregnant women in the Asia population (15), which is not conducive for Chinese pathologists and obstetricians to obtain information and compare it to other countries (16). At the same time, evidence in different classifications has supported the relationship between placental lesions and preterm birth (17, 18). However, there is limited evidence about the relationship between single placental pathology or superimposed pathology with the outcome in preterm births with or without premature rupture of membranes (PROM), and thus, further exploration is needed (19).

Therefore, this study aims to retrospectively explore data from our hospital and discuss the relationship between the single or co-occurrence of MVM and AI with outcomes such as gestational age (GA), birth weight, neonatal respiratory distress syndrome (RDS), and intraventricular hemorrhage (IVH) in literature (14, 19, 20). Finally, through the above research, we hope to highlight the application value of standardized placental pathological interpretation in diagnosing and treating diseases.

## Materials and methods

### Study population

**Figure 1:**
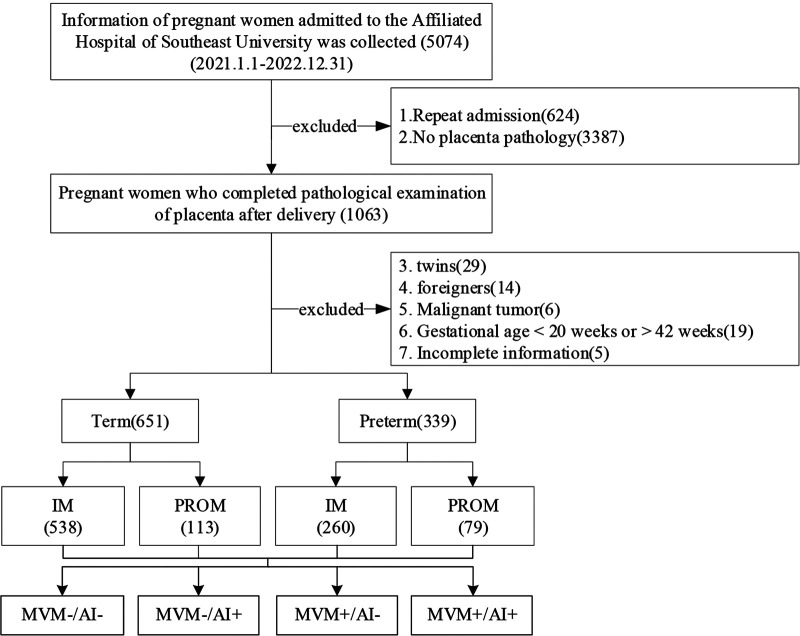
Flow chart of the study cohort screening in Zhongda Hospital Affiliated to Southeast University from Jan 2021 to Dec 2022. IM, Intact membranes; PROM, Premature rupture of membranes; MVM, Maternal vascular malperfusion; AI, Acute infection/inflammation.

A single-center, retrospective study was performed at Zhongda Hospital of Southeast University in Nanjing, Jiangsu Province, China. We searched the records of 5,074 pregnant women who were admitted between 01. January. 2021 and 31. December. 2022. 4084 pregnant women who did not meet the requirements were excluded: (1) Repeated hospitalizations for other diseases prior to delivery, (2) No placental pathology, (3) twins, (4) foreigners, (5) Malignant tumor, (6) pregnant female <20 weeks or >42 weeks of gestation, and (7) women with incomplete information. Finally, the cohort included 990 singletons with a gestational age of 20 + 0 to 41 + 6 weeks (Figure 1). The study was approved by the hospital’s medical ethical Committee (Ethics Number: 2021ZDSYLL338-P01).

### Clinical data collection

The following maternal clinical data were collected from the Electronic medical record system: Pre-pregnancy BMI, Education, Age, Delivery mode, Diabetes, and Hypertension. The neonatal data that was recorded from the Electronic medical record system included: GA, birth weight, Neonatal intensive care unit (NICU), 1-minute Apgar score and 5-minute Apgar score, Small for gestational age (SGA), large for gestational age (LGA), gender, RDS and IVH. In addition, We calculated birth weight Z-scores for different sexes using L, M, and S values from WHO reference data (21).

### Diagnosis of MVM and AI

The diagnosis of MVM, according to the results presented by Amsterdam and Redline, mainly includes (decidual arteriopathy, villus infarction, increased Syncytial Knots, perivillous fibrin deposition, and intervillous fibrin deposition), MVM is used as a whole, not graded (8, 22)—AI including histologic chorioamnionitis (HCA), vasculitis, and funisitis. No HCA and HCA stage I were defined as mild, and stage II and III were defined as severe, with further grouping for severe HCA (Supplementary Table S1) (23).

### Groups

According to MVM and AI, the two placental lesions were divided into four groups: None of the MVM nor AI (MVM-/AI-), AI alone (MVM-/AI+), MVM alone (MVM+/AI-), co-occurrence of MVM and AI (MVM+/AI+).

## Statistical methods

Maternal and infant information was grouped according to preterm birth and rupture of membranes. Categorical variables were analyzed with chi-square tests. After Continuous variables were tested for normal distribution and homogeneity of variance, using ANOVA or Kruskal-Wallis test, as appropriate. *P* values were corrected for multiple testing using the Bonferroni procedure. Categorical variables are expressed as quantities and percentages. The continuous variables were expressed as mean ± standard deviation if normally distributed and as median (P25, P75) if not normally distributed. Linear regression was used to simulate the GA and birth weight Z-score (MVM-/ AI- as a reference, compare the cases of MVM-/ AI+, MVM+/ AI-, and MVM+/ AI+) and expressed as *β*, *P*. We conducted logistic regression analyses to determine the associations between placental findings and neonatal RDS and IVH. Results were expressed as Odds Ratio (OR) 95% confidence intervals (CI). The covariates selected *a priori* mainly included age, college education, and pre-pregnancy BMI. During the discussion of RDS and IVH, the GA covariate will be added as an adjustment. R (Version 4.2.2) software was used for the above statistical analysis, and *P* < 0.05 was considered statistically significant.

## Results

From 01. January. 2021 to 31. December. 2022, clinical information on 990 pregnant women and newborns was assessed. Pregnant women were divided into four groups based on preterm birth and premature rupture of membranes. There were 651 term pregnant women, including 538 with intact membranes and 113 with PROM. There were 339 preterm pregnant women, including 260 with intact membranes and 79 with PPROM (Figure 1).

Among the four groups, low education was more likely to occur in preterm pregnancy with intact membranes (84%, 85.8%, 60%, and 74.7%, respectively; *P *< 0.001). Preterm pregnancy with intact membranes was more likely to develop hypertension (chronic hypertension 3.5%, 0.9%, 7.3%, 1.2%, and gestational hypertension 7.1%, 1.7%, 13.5%, 2.5%, respectively; *P* < 0.001). Newborns in preterm pregnancy with intact membranes were more likely to have a 1-minute Apgar score < 7 (1.5%, 0.0%, 26.2%, 10.1%, respectively; *P* < 0.001), 5-minute Apgar score < 7 (1.1%, 0.0%, 20.0%, 7.6%, respectively; *P* < 0.001). Newborns in preterm pregnancy with intact membranes were more likely to have RDS (0.7%, 0.0%, 31.9%, 31.6%, respectively; *P* < 0.001) and BPD (0.0%, 0.0%, 8.1%, 6.3%, respectively; *P* < 0.001) and the IVH (0.9%, 0.9%, 20.0%, 17.7%, respectively; *P* < 0.001) adverse outcomes. Among the four groups, Placental lesions are more likely to occur in preterm infants with intact membranes, including decidual arteriopathy (2.6%, 3.5%, 9.6%, 5.1%, respectively; *P* = 0.010) and placental infarction (15.4%, 22.1%, 13.5%, 5.1%, respectively; *P* < 0.01). However, FIRS (1.5%, 1.8%, 2.7%, 6.3%, *P* = 0.049) and severe HCA (21.9%, 26.5%, 23.1%, 44.3%, respectively; *P* < 0.001) were higher in pregnant women with PPROM. Advanced Syncytial knots (65.1%, 61.1%, 58.8%, 65.8%, respectively; *P* = 0.34), Perivillous fibrin deposition (58.4%, 58.4%, 49.2%, 50.6%, respectively; *P* = 0.07) and Intervillous fibrin deposition (2.8%, 4.4%, 3.1%, 1.3%, respectively; *P* = 0.634) were not statistically significant among the four groups (Table 1).

**Table 1:** Maternal and infant clinical features and placental pathology. Mean (P25, P75) or (*n*, %) indicates results.

	Term			Preterm			* *
	TOTAL (*N* = 651)	IM (*N* = 538)	PROM (*N* = 113)	TOTAL (*N* = 339)	IM (*N* = 260)	PPROM (*N* = 79)	*P* value
**Maternal characteristics**							
Pre-pregnancy BMI	21.6 (19.8–23.9)	21.6 (19.9–24.0)	21.1 (19.3–23.1)	21.8 (19.8–25.0)	22.1 (19.9–25.3)	21.4 (19.6–24.0)	0.100
College Education	549 (84.3)	452 (84.0)	97 (85.8)	215 (63.4)	156 (60.0)	59 (74.7)	<0.001
Age	30 (28–33)	30 (28–33)	30 (27–32)	30.7 (27–34)	31 (27–34)	30 (27–32)	0.123
Delivery mode							<0.001
Vaginal	117 (18.0)	91 (16.9)	26 (23.0)	123 (36.3)	79 (30.4)	44 (55.7)	
Midwifery	13 (2.0)	11 (2.0)	2 (1.8)	7 (2.1)	4 (1.5)	3 (3.8)	
Cesarean	521 (80.0)	436 (81.0)	85 (75.2)	209 (61.7)	177 (68.1)	32 (40.5)	
Diabetes							0.407
Preexisting	10 (2.9)	9 (1.7)	1 (0.9)	9 (2.7)	9 (3.5)	0 (0.0)	
Gestational	126 (19.4)	106 (19.7)	20 (17.7)	65 (19.2)	48 (18.5)	17 (21.5)	
Hypertension							<0.001
Preexisting	20 (3.1)	19 (3.5)	1 (0.9)	20 (5.9)	19 (7.3)	1 (1.2)	
Gestational	41 (6.3)	38 (7.1)	3 (1.7)	37 (10.9)	35 (13.5)	2 (2.5)	
**Neonatal characteristics**							
NICU	89 (13.7)	76 (14.1)	13 (11.5)	229 (67.6)	175 (67.3)	54 (68.3)	<0.001
1Apgar < 7	8 (1.2)	8 (1.5)	0 (0.0)	76 (22.4)	68 (26.2)	8 (10.1)	<0.001
5Apgar < 7	6 (0.9)	6 (1.1)	0 (0.0)	58 (17.1)	52 (20.0)	6 (7.6)	<0.001
Growth mode							<0.001
SGA	50 (7.7)	58 (10.8)	9 (8.0)	34 (10.0)	32 (12.3)	2 (2.6)	
LGA	67 (10.3)	45 (8.4)	5 (4.4)	4 (1.2)	4 (1.5)	0 (0.0)	
Gender							0.024
Male	339 (52.1)	286 (53.2)	53 (46.9)	204 (60.2)	152 (58.5)	52 (65.8)	
Female	312 (47.9)	252 (46.8)	60 (53.1)	131 (38.6)	104 (40.0)	27 (34.2)	
RDS	4 (0.6)	4 (0.7)	0 (0.0)	118 (34.8)	83 (31.9)	25 (31.6)	<0.001
BPD	0 (0.0)	0 (0.0)	0 (0.0)	26 (7.7)	21 (8.1)	5 (6.3)	<0.001
IVH	6 (0.9)	5 (0.9)	1 (0.9)	66 (19.5)	52 (20.0)	14 (17.7)	<0.001
**Placenta pathology**							
MVM	529 (81.3)	441 (82.0)	87 (77.0)	255 (75.2)	197 (75.8)	57 (72.1)	0.060
Vasculopathy	18 (3.0)	14 (2.6)	4 (3.5)	29 (8.6)	25 (9.6)	4 (5.1)	<0.001
Infarct	108 (16.6)	83 (15.4)	25 (22.1)	39 (11.5)	35 (13.5)	4 (5.1)	0.010
Syncytial knots	419 (64.4)	350 (65.1)	69 (61.1)	205 (60.5)	153 (58.8)	52 (65.8)	0.340
Perivillous fibrin	380 (58.4)	314 (58.4)	66 (58.4)	168 (49.6)	128 (49.2)	40 (50.6)	0.070
Intervillous fibrin	20 (3.1)	15 (2.8)	5 (4.4)	9 (2.7)	8 (3.1)	1 (1.3)	0.634
FIRS	10 (1.5)	8 (1.5)	2 (1.8)	12 (3.5)	7 (2.7)	5 (6.3)	0.049
Mild HCA	503 (77.3)	420 (78.1)	83 (73.5)	244 (72.0)	200 (76.9)	44 (55.7)	<0.001
Severe HCA	148 (22.7)	118 (21.9)	30 (26.5)	95 (28.0)	60 (23.1)	35 (44.3)	

SGA, small-for-gestational-age; LGA, large-for-gestational-age; NICU, neonatal intensive care Unit; RDS, neonatal respiratory distress syndrome; IVH, intraventricular hemorrhage; BPD, bronchopulmonary dysplasia; MVM, maternal vascular malperfusion; HCA, histological chorioamnionitis; FIRS, Fetal inflammatory response syndrome; IM, Intact membranes; PPROM, preterm premature rupture of membranes. *P-*values are for IM, PROM in term, and IM, PPROM in preterm four groups comparison. Furthermore, the Bonferroni test procedure-corrected *P* values between sample pairs are shown. A corrected *P* value of <0.008 was considered statistically significant.

**Figure 2:**
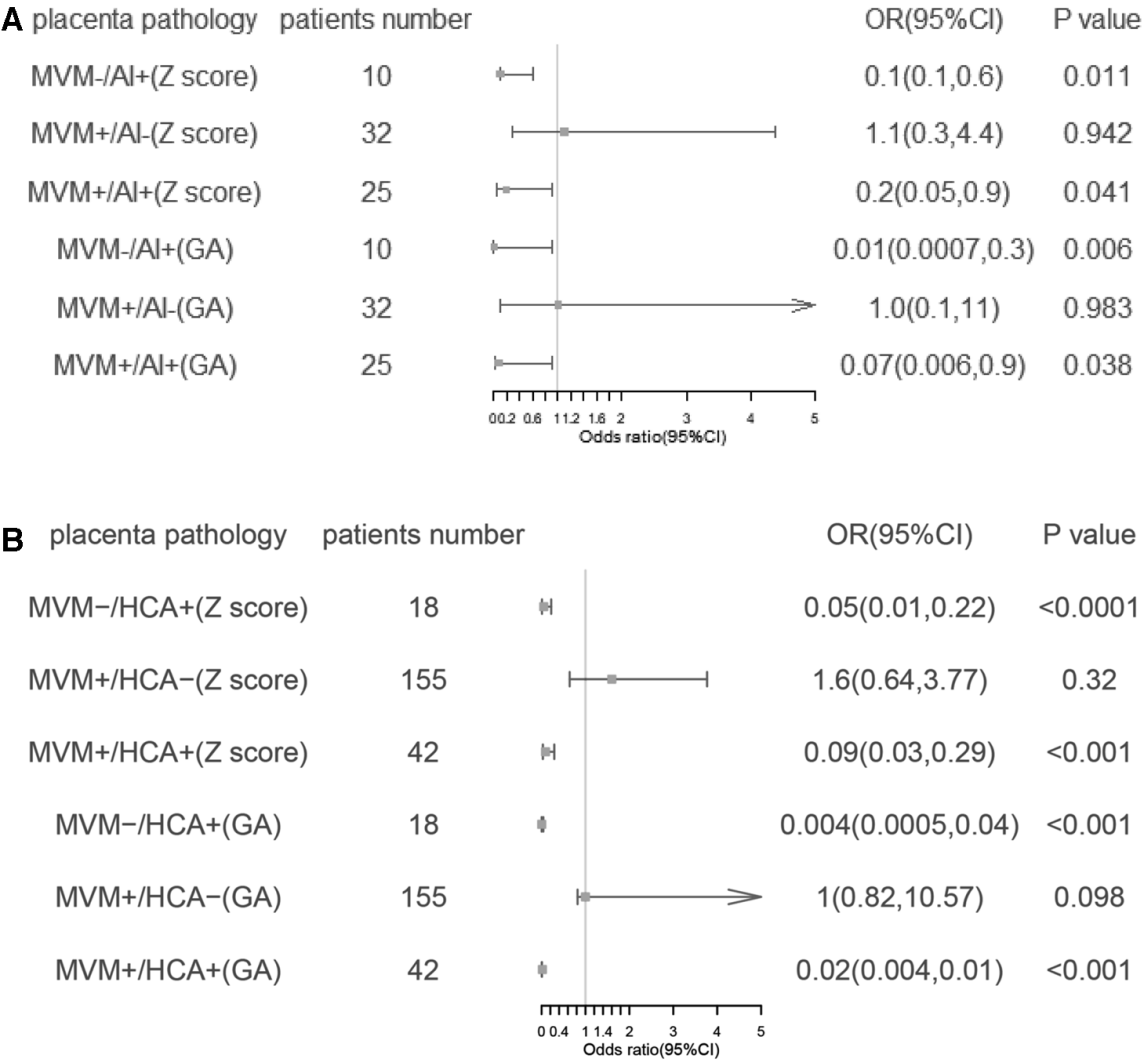
Forest map of Z score and GA in PPROM (**A**) and premature births with intact membranes (**B**). GA, gestational age; Z-score, birth weight z-score; PPROM, preterm premature rupture of membranes. MVM, maternal vascular malperfusion; AI, acute infection/inflammation; MVM-/AI- is the referent.

After adjusting the covariates of age, college education, and pre-pregnancy BMI, the effects of MVM-/AI-, MVM-/AI+, MVM+/AI- and MVM+/AI+ on GA and Z-score of the newborns were explored.In reference to MVM-/AI-, MVM−/AI+ at term was paradoxically associated with, on average, slightly longer gestations (difference 0.6 weeks, *p *= 0.02). MVM-/AI+ and MVM+/AI+ were associated with shorter gestational weeks of newborns (Adjusted GA −4.7, and−3.0, both *P* < 0.001). MVM-/AI + and MVM+/AI+ with intact membrane (Adjusted GA −5.4, *P* < 0.001 and −3.8, *P* = 0.001) PPROM (Adjusted GA −4.3, *P* = 0.006 and −2.6, *P* = 0.038) may result in a shorter gestational age in preterm birth. In reference to MVM-/AI-, MVM+/AI+ was associated with lower birth weight for preterm birth (adjusted birth weight Z-score −2.6 and −1.8, both *P* < 0.001). In preterm infants, MVM-/AI+ and MVM+/AI+ with intact membrane (adjusted birth weight Z-score −3.0, and −2.4, both *P* < 0.001) versus PPROM (adjusted birth weight −2.4, *P* = 0.011 and −1.6, *P* = 0.041) were associated with lower birth weight (Table 2, Figures 2A, B).

**Table 2:** The relationship between GA, birth weight Z-score and placenta pathology.

	GA(*β*, *P*)								z-score(*β*, *P*)							
	Term		Preterm		IM		PPROM		Term		Preterm		IM		PPROM	
	*β*	*P*	β	*P*	β	*P*	β	*P*	β	*P*	β	*P*	β	*P*	β	*P*
MVM - /AI-	Ref.		Ref.		Ref.		Ref.		Ref.		Ref.		Ref.		Ref.	
MVM -/ AI+	0.7	0.008	−5.0	<0.001	−5.8,	<0.001	−4.3	0.005	−0.2	0.440	−2.7	<0.001	−3.1	<0.001	−2.4	0.011
MVM +/ AI -	0.1	0.281	0.8	0.196	0.9	0.155	0.3	0.793	−0.12	0.290	0.3	0.414	0.4	0.394	0.3	0.677
MVM+/ AI+	0.6	<0.001	−3.1	<0.001	−4.1	<0.001	−2.2	0.079	0.01	0.966	−1.9	<0.001	−2.6	<0.001	−1.2	0.113
	aβ	*P*	aβ	*P*	aβ	*P*	aβ	*P*	aβ	*P*	aβ	*P*	aβ	*P*	aβ	*P*
MVM - /AI-	Ref.		Ref.		Ref.		Ref.		Ref.		Ref.		Ref.		Ref.	
MVM -/ AI +	0.6	0.021	−4.7	<0.001	−5.4	<0.001	−4.3,	0.006	−0.2	0.566	−2.6	<0.001	−3.0	<0.001	−2.4	0.011
MVM +/ AI -	0.1	0.428	0.9	0.144	1.1	0.100	−0.02	0.984	−0.1	0.312	0.3	0.377	0.4	0.327	0.1	0.942
MVM +/ AI +	0.5	<0.001	−3.0	<0.001	−3.8	<0.001	−2.6	0.038	0.03	0.833	−1.8	<0.001	−2.4	<0.001	−1.6	0.041

The continuous dependent variables are GA and Z-score; β, no adjust; aβ, adjusted for pre-pregnancy BMI, Age, college education, MVM, maternal vascular malperfusion; AI, acute infection/inflammation; Ref., referent.

In this study, a higher risk of RDS in newborns in MVM+/AI + was observed only in preterm populations with OR 2.2 (95%CI 1.03, 4.9); however, this association was not observed after further adjustment for gestational age at birth as a covariate (Table 3). No association with IVH and RDS was observed in comparisons of MVM-/AI+, MVM+/AI-, and MVM+/AI+, regardless of intact membranes or PPROM. However, after adjusting for pre-pregnancy BMI, Age, college education, and GA, we observed a significant increase in MVM-/AI+, MVM+/AI-, and MVM+/AI+ (Adjusted OR 0.9, 1.5, and 1.8) in RDS. Although there was no statistically significant difference in disease risk, the risk of disease occurrence showed an increasing trend. This trend was observed in preterm women with intact membranes (adjusted for OR 0.9, 1.8, 1.8) and in women with PPROM (adjusted for OR 0.2, 0.4, and 1.0).

**Table 3:** Relationship between RDS and IVH and placental pathology.

	RDS (OR 95%CI)			IVH (OR 95%CI)		
	Preterm	IM	PPROM	Preterm	IM	PPROM
	OR 95%CI	OR 95%CI	OR 95%CI	OR 95%CI	OR 95%CI	OR 95%CI
MVM - /AI -	Ref.	Ref.	Ref.	Ref.	Ref.	Ref.
MVM -/ AI +	1.2 (0.4, 3.4)	1.2 (0.3, 4.3)	0.8 (0.1, 1.0)	0.6 (0.2, 2.0)	0.9 (0.2, 3.3)	n.a.
MVM +/ AI -	1.4 (0.7, 2.8)	1.7 (0.8, 3.8)	0.5 (0.1, 1.0)	0.8 (0.4, 1.8)	0.7 (0.3, 1.6)	1.0 (0.3, 42.5)
MVM+/AI +	2.2 (1.03, 4.9)	2.2 (0.8, 5.7)	3.0 (0.7, 15.5)	1.1 (0.5, 2.7)	0.8 (0.3, 2.2)	1.0 (0.6, 82.2)
	aOR 95%CI	aOR 95%CI	aOR 95%CI	aOR 95%CI	aOR 95%CI	aOR 95%CI
MVM - /AI -	Ref.	Ref.	Ref.	Ref.	Ref.	Ref.
MMV -/ AI +	0.8 (0.3, 2.4)	0.9 (0.2, 3.3)	0.2 (0.0, 1.0)	0.5 (0.1, 1.6)	1.0 (0.2, 3.5)	n.a.
MVM +/ AI -	1.5 (0.7, 3.0)	1.8 (0.8, 4.1)	0.4 (0.1, 1,0)	0.9 (0.4, 1.9)	0.7 (0.3, 1.7)	1.0 (0.2, 4.1)
MVM+/ AI+	1.8 (0.8, 4.0)	1.8 (0.7, 4.8)	1.0 (0.3, 1.0)	0.9 (0.4, 2.3)	0.8 (0.3, 2.2)	1.0 (0.2, 4.2)

The dependent variables are RDS and IVH; n.a., The number of cases is too small to be counted; CI, confidence interval; aOR, adjusted odds ratio, namely adjusted for gestational age (as a continuous variable), pre-pregnancy BMI, Age, college education; RDS, respiratory distress syndrome; IVH, intraventricular hemorrhage; IM, intact membranes; PPROM, preterm premature rupture of membranes; Ref., referent.

## Discussion

Proper placental function is essential for exchanging and transporting nutrients and waste between mothers and newborns. This study investigated the relationship between MVM, AI alone, or with co-occurrence of adverse outcomes in pregnant women in China and further investigated the relationship between placental disease and preterm birth with or without PPROM.

The following key information was obtained through this retrospective study. First, although no effect of MVM was observed on either outcome, pathological features of MVM and AI were dominant in preterm birth with intact membranes and PPROM. It is suggested that MVM and AI are two important subtypes of placental lesions. This partly explains the differences between the different types of preterm birth. Second, the presence of MVM or AI alone and co-occurrence was associated with shorter GA and lower birth weight in newborns. The coexistence of MVM and AI may be a unique pathological form that needs attention, providing a unique perspective and thought for explaining the pathological mechanism of neonatal diseases. Finally, the presence of MVM or AI alone and co-occurrence in RDS reflects an increasing trend of disease risk, suggesting that different degrees of placental lesions may be closely related to the risk of disease occurrence. Predicting the severity of diseases from the perspective of placental lesions may be possible and may provide theoretical support for implementing disease intervention and reducing the risk of short—and long-term poor prognosis of diseases.

In this study, AI alone, MVM alone, MVM, and AI co-occurrence were associated with lower birth weight and shorter GA in preterm birth. Karen Mestan’s study confirmed that placental infection/inflammation is related to postpartum fetal growth and may be an effective indicator for predicting postpartum fetal growth failure (24). The inflammatory cascade induced by AI is an essential mechanism of preterm birth (25–27). Microorganisms are recognized by innate immune system monitoring receptors (TLRs), leading to the activation of pro-inflammatory transcription factor nuclear factor Kappa-B (NF-kB) and the production of downstream pro-inflammatory cytokines (27–29). Microbial endotoxins and pro-inflammatory cytokines stimulate the production of prostaglandins, other inflammatory mediators, and stroma-degrading enzymes. Prostaglandins stimulate uterine contraction, and degradation of the extracellular matrix of the fetal membrane can lead to PPROM (25). This may explain why the incidence of HCA and FIRS in this study’s PPROM population was higher than in other groups.

The diagnosis of MVM does not depend on any single placental finding but rather on a series of findings involving primary changes in the maternal decidual vasculature of the mother and/or secondary changes in the villous parenchymal (30). MVM is also a pathological phenomenon in preeclampsia, stillbirth, fetal growth restriction, and other diseases (31–33). Although MVM did not differ among the four groups, co-occurrence of MVM and AI was associated with shorter GA and weight loss in our study, suggesting that MVM remains a significant placental lesion. It has been reported that direct detection of placenta-related biomarkers—continuous cycle maternal placental growth factor can lead to a high proportion of patients with a poor perinatal prognosis, which helps test disease treatment strategies to improve clinical prognosis (34).

As a result, pathologists will likely encounter lesions of AI and MVM in daily practice and provide useful information about the underlying causes of preterm birth (4). In response to the emergence of AI, some guidelines recommend using antibiotics before pregnancy to reduce the risk of developing AI if suspected (35, 36). While there is no specific treatment for MVM, potential management options include optimizing maternal health and paying attention to cardiovascular status, glucose tolerance, and weight (37–40). Other possible interventions include susceptibility assessment, uterine artery doppler, early placental ultrasound in the third trimester, drug therapy, and early delivery in subsequent trimesters (41–43). At the same time, it has been suggested that pathologists should be aware of the emergence and evolution of MVM and associated pathological lesions and strive to use standardized diagnostic templates to identify placental lesions (30). Because accurate identification has potential clinical relevance/prognostic implications for mothers and children, consistent reporting may advance our deeper understanding of placental lesions to improve management and identify potential treatments (44).

## Limitations and strengths

This study’s limitations are as follows: first, the present study is a single-center retrospective study, and evidence from a large-sample multi-center prospective study is required to determine whether the findings apply to the women in China. Second, there is still a gap in the interpretation of placenta pathology in our hospital compared with other countries. Although we also supplemented the data by integrating indicators from other literature, standardized diagnostic templates are still needed to identify placental lesions in subsequent clinical diagnosis and treatment to enhance the general value of clinical reports and identify potential therapies.

The strengths of this study are as follows. First, data in this study is from the Chinese population, which is of particular reference value for comparing different races to those previously documented. Second, the present study provides evidence on the relationship between the appearance of placental pathology alone or co-occurrence and guides subsequent researchers to design and further explore the research direction. Thirdly, through this study, we also realized the non-standardization and application limitations of the existing placental pathology interpretation and made timely improvements. The subsequent standardized diagnosis of placenta pathology promotes the universality of placental pathological interpretation reports. In clinical application, it can guide the diagnosis and treatment of diseases more scientifically.

## Conclusion

MVM and AI are two important subtype placental lesions associated with adverse outcomes in preterm infants. AI or MVM alone and a combination of both lesions may be associated with an increased risk of certain diseases. Therefore, it is of great significance to improve the standard of placental pathology interpretation to standardize its general application, guide disease diagnosis, and make the diagnosis and treatment plan scientifically.

## Data Availability

The original contributions presented in the study are included in the article/**Supplementary Material**, further inquiries can be directed to the corresponding author.
