# Supplementary material for: Relationship between placental pathology and neonatal outcomes
Source: Front Pediatr. 2023 Jun 15;11:1201991. doi: 10.3389/fped.2023.1201991 (PMC10309182; doi:10.3389/fped.2023.1201991)
Supplement: Supplementary file 1 [file Table1.docx]

| **Supplementary Table 1** Definition of placental pathology lesions[8, 22] | |
| --- | --- |
| **Term** | **Definition** |
| Maternal vascular malperfusion (MVM) | Trophoblastic dysfunction and defective arterial remodeling. |
| Acute infection/inflammation (AI) | Histological chorioamnitis and Fetal Inflammatory Responses. |
| Histological chorioamnitis (HCA) | Stage 1: acute subchorionitis or chorionitis;  Stage 2: polymorphonuclear leukocytes extend into amnion and/or fibrous chorion;  Stage 3(necrotizing chorioamnionitis): karyorrhexis of polymorphonuclear leukocytes, amniocyte necrosis, and/or amnion basement membrane hypereosinophilia. |
| Fetal Inflammatory Response (FIRS) | Stage 1: umbilical phlebitis or chorionic vasculitis;  Stage 2: involvement of the umbilical vein and one or two umbilical arteries. |
| Decidual Arteriopathy | The elements include acute atherosis, fibrinoid necrosis with or without foam cells, chronic perivasculitis, absence of spiral artery remodeling, mural hypertrophy, arterial thrombosis, and persistence of intramural endovascular trophoblast in the third trimester |
| Syncytial knot increased | knots on more than 33% of villi may be regarded as increased. |
| Villi infarction | The collapse of more than five villi interstitial Spaces with scattered trophoblast degeneration and ischemic necrosis of villi. |
| Perivillous fibrin deposition | Irregular zones of fibrinoid material tightly encase the entrapped villi. A small amount of perivillous fibrin is acceptable in the upper third of the placental parenchyma. |
| Intervillous fibrin deposition | An increased percentage (3% is the upper limit) of small foci of fibrinoid material within or adjacent to the villi. |
